# Disease, and Poverty

**Published:** 1858-11

**Authors:** 


### DISEASE, AND POVERTY,

And widowhood, with young orphan children, often meet in one household in this great city. A drear assembly that, in cheerless garrets, and more miserable cellars, in mid-Winter too! Who will help, from a three-cent stamp and upwards? The hapless widow, aided a year ago by a lady subscriber from the South, grows more steadily helpless. Will our out-spoken Presbyterian Cotton-planter, from the broad fields of Alabama, send a liberal check to us, payable to the order of Rev. CHARLES C. DARLING, No. 4 Rutherford Place, to be distributed according to the judgment of Mrs. E. H., who is a manager of the society for aiding destitute widows with young children? As a single dollar lost, is the loss of a week's aid to some wretched family, up to five dollars may be sent in bank-bills; but over five, in checks, drafts, or orders. Dollar gold pieces are slipped out of a letter with marvellous ease, by persons of leisure, with the aid of a penknife, making a neat tiny slit in the edge of the envelope.

